# Transforming health equity through academic integration: ripple effects of high-impact practices in CBPR with refugee-origin Montagnard youth

**DOI:** 10.3389/fpubh.2025.1672085

**Published:** 2026-01-14

**Authors:** Sharon D. Morrison, S. Sudha, Andrew Young, H’Yua Adrong

**Affiliations:** 1 Department of Public Health Education, University of North Carolina Greensboro, Greensboro, NC, United States; 2 Department of Human Development and Family Studies, University of North Carolina Greensboro, Greensboro, NC, United States; 3 Montagnard/Asian Community Disparities Research Network, Greensboro, NC, United States; 4 Montagnard Association of North Carolina, Greensboro, NC, United States

**Keywords:** Community-Based Participatory Research, health equity, high-impact practices, refugee-origin youth, ripple effects

## Abstract

This community case study describes an innovative model of academic integration, the integration of university-based high-impact practices (HIPs) with Community-Based Participatory Research (CBPR)—an approach rooted in co-learning, shared power, and mutual benefits—to advance health equity for low-income Asian American refugee-origin communities (LIAACRO) in central North Carolina. The Montagnard Hypertension Project (MHyP), a community-initiated collaboration, embedded five HIPs, service learning, undergraduate research, diversity/global learning experiences, internships, and capstone projects, within a CBPR framework to bring community partners into academic spaces as cultural experts and co-researchers. This academic integration enabled Montagnard college students (MCS) to access institutional resources, gain research competencies, and lead culturally responsive health outreach. Montagnards, an indigenous people from Vietnam’s Central Highlands, experience persistent invisibility in U.S. health and social systems due to data aggregation and the Asian American “model minority” myth. Through the MHyP, the MCS leveraged their cultural knowledge and lived experiences to co-develop culturally and linguistically appropriate research protocols, design and implement community health promotion initiatives, facilitate intergenerational dialogue, and document traditional practices. Using a ripple effects framework, we retrospectively assess multilevel changes (individual, community, and institutional) that extend beyond the project’s scope. Findings demonstrate how carefully and strategically integrating HIPs can strengthen CBPR’s principles of co-learning, shared leadership and decision-making, empowerment, and mutual benefit. The model offers a replicable pathway for public health researchers and practitioners to engage marginalized communities in ways that promote educational advancement, community values and empowerment, and institutional transformation. This case contributes to the field by showing how academic institutions and public health practitioners can reimagine and successfully mobilize institutional resources, including curricula, internal funding, and campus spaces, to support sustainable, equity-driven community engagement.

## 1 Introduction

Low-income Asian American communities of refugee origin (LIAACRO) face a complex web of challenges that traditional public health approaches often fail to address effectively. Despite experiencing poverty rates significantly higher than those of other Asian Americans, these communities remain invisible in institutional data systems due to data aggregation and the widespread “myth” that falsely depicts all Asian Americans as high-income earners (1–3). This invisibility creates a critical gap in public health efforts, namely the lack of targeted interventions for LIAACRO, whose unique needs stay hidden behind aggregated Asian statistics. Public health practitioners working with marginalized refugee populations may know this issue. Standard top-down approaches that depend on external academic expertise and pre-designed interventions tend to be especially ineffective when working with communities facing intersecting challenges like poverty, language barriers, cultural adaptation stress, and systemic marginalization (4–6). Historical trauma, discrimination, and ongoing socioeconomic issues foster distrust of external interventions, making genuine community engagement vital for building relationships that lead to meaningful health improvements (7).

Community-Based Participatory Research (CBPR) is a collaborative approach that involves community members and researchers equally throughout the research process (8, 9). Grounded in Freire’s pedagogy of dialogue and critical consciousness, CBPR engages communities as both co-learners and co-leaders in a transformative process that connects reflection to collective local action for health equity (10–12). Unlike conventional research, which often occurs without community input, CBPR emphasizes equitable collaboration. It promotes shared power and decision-making, allowing communities to identify and prioritize problems and devise solutions (13, 14). It is a more inclusive approach to public health research with refugee populations whose lived experiences are often hidden and marginalized (15, 16). CBPR, as a strengths-based approach, challenges deficit narratives by emphasizing community assets and resilience (17, 18). As a culturally grounded framework, it counteracts the model minority myth, unmasks disparities, unpacks disaggregated data, and debunks false expectations about the resources and educational support available to refugee families (3, 19). CBPR aligns well with socioecological models that recognize multilevel influences on health and wellbeing (20, 21).

LIAACRO youth often act as cultural brokers, skillfully managing bicultural identities while managing family caregiving responsibilities and economic pressures alongside their educational pursuits (22, 23). However, higher education systems that prioritize traditional college student experiences often overlook or underestimate these overlapping roles and challenges. CBPR, as a participatory approach, emphasizes and amplifies LIAACRO youth voice and agency (24). Its core principles of knowledge co-creation, cyclical and iterative engagement, and empowerment support inclusive research that respects community-defined priorities, addresses power differences, and encourages collaborative problem-solving (9, 25–27). For LIAACRO youth, this involves building long-term, trusted relationships that increase capacity (12, 17). These components are crucial for advancing rigorous and responsive public health research that explores complex and intersectional experiences and everyday realities.

The Association of American Colleges and Universities (AAC&U) defines High-Impact Practices (HIPs) as educational strategies that significantly improve student learning, boost retention, and encourage engagement (28). These practices, such as undergraduate research, service-learning, diversity and global education, internships, and capstone experiences, are especially beneficial for first-generation college students and individuals from historically underrepresented groups (28, 29). HIPs are based on experiential teaching methods that highlight real-world relevance, meaningful faculty-student interactions, significant time commitments, exposure to diversity, and deliberate reflection (30, 31). While individual HIPs have positively affected learners, combining multiple HIPs can further enhance learner success (30).

When integrated with CBPR, HIPs become powerful tools for co-learning and co-creating knowledge, aligning with educational goals. For example, a semester-long public health class project incorporated CBPR principles, where students collaborated with communities to develop knowledge and health education materials (32). CBPR’s focus on collaborative inquiry and empowerment complements HIPs’ educational objectives of deep learning and inclusive practice (25, 26). For marginalized youth, including those from LIAACRO, integrating HIPs with CBPR offers structured pathways to use practical knowledge and the lived experiences of navigating bicultural identity and caregiver roles. Youth can demonstrate expertise and help improve research rigor and quality, affirming the value of participatory scholarship (31, 33, 34). HIPs in CBPR enable community access to institutional resources that might otherwise be out of reach.

This community case study highlights an innovative model of integrating HIPs into CBPR to promote health equity for LIAACRO in central North Carolina. The Montagnard Hypertension Project (MHyP), a community-initiated collaboration representing the core of this model, successfully integrated five university-based HIPs with a CBPR approach. Three key goals were to (1) engage community members with academic researchers and institutional settings, (2) empower Montagnard college students (MCS), youth from an indigenous refugee community in Vietnam’s Central Highlands, as researchers, and (3) generate ripple effects leading to both intended and unintended multilevel outcomes (35). Despite nearly 40 years since resettlement, Montagnards remain neglected and underserved by U.S. health and social systems, hidden within Asian American “model minority” statistics, and overlooked for targeted public health efforts (1, 3, 35). HIPs offer academic pathways recognizing MCS as bridges between traditional cultural knowledge and modern research frameworks (33). Integrating HIPs within CBPR provides a novel way to support the professional development of first-generation MCS, who face intersecting challenges of cultural invisibility, household poverty, and family responsibilities. At the same time, this approach reduces access barriers by directing academic financial resources and spaces toward communities that have historically been excluded from such opportunities (34, 35). The following sections outline the ripple effects framework, the MHyP phases, multilevel outcomes, and a discussion of the implications, limitations, and conclusions. We aim to provide practical tools for applying similar approaches that honor community knowledge, ownership, and cultural protocols in public health practice with refugee populations across global contexts.

## 2 Methods

### 2.1 Guiding framework

**Figure 1:**
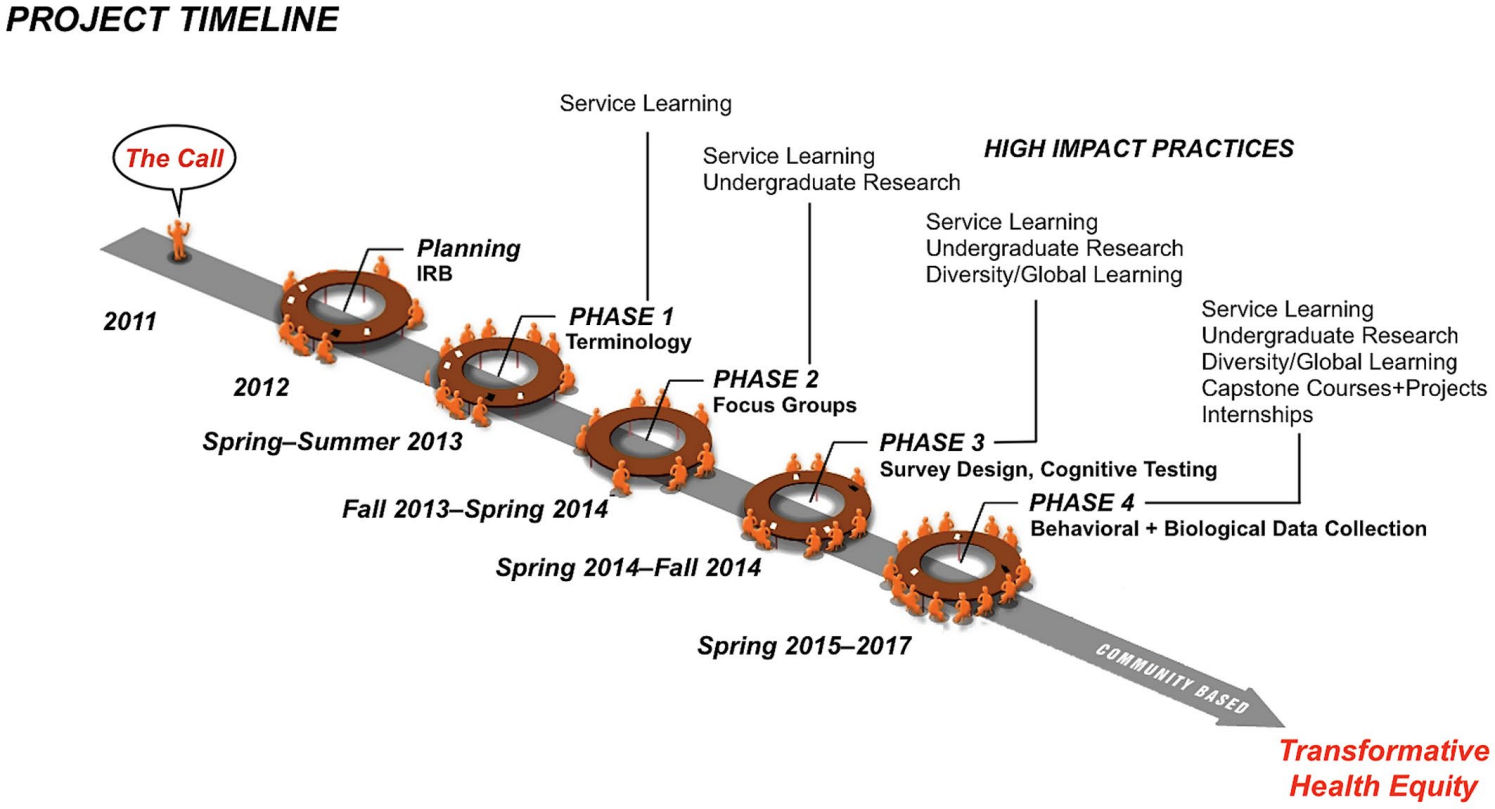
Montagnard Hypertension Project (MHyP) timeline and phases.

In community and youth development, ripple effects refer to the indirect and often unintended outcomes of programs that go beyond their immediate goals, leading to cascading changes at various levels and over time (36–39). As a repeatable framework, ripple effects help public health researchers and practitioners document both planned and unanticipated consequences of community engagement. Professionals can track how individual changes contribute to broader community transformation and influence institutional policies, while also identifying pathways toward health equity that extend beyond project scopes and timelines (39) (Figure 1). Importantly, not all ripple effects are positive. Cho and Salmon (40) identified harmful unintended outcomes such as obfuscation, boomerang effects, and social norming that are particularly relevant to HIPs and CBPR with marginalized communities. For example, service-learning or capstone projects may unintentionally produce culturally mismatched materials when the community context is misunderstood (obfuscation) or reinforce stigma through judgmental messaging (social norming). CBPR with integrated HIPs may also reproduce academic hierarchies if community voices are not equally included (social norming) or impose opportunity costs when community time and resources are used without clear reciprocity. Guta et al. (41) and Damon et al. (42) highlight that CBPR can create emotional labor for participants, especially when academic schedules and community rhythms clash. Without meaningful reflection and cultural sensitivity, the HIPs-CBPR approach risks replicating institutional power structures under the guise of collaboration (27).

We use the ripple effects framework to analyze the multi-level impact of the multi-phase MHyP retrospectively, based on the following key question: *How did the strategic integration of HIPs for Montagnard college students (MCS) involved in a CBPR project lead to changes at the individual, community, and institutional levels?*

For global public health researchers and practitioners, this case study offers a replicable framework for involving marginalized youth as cultural experts while building sustainable community capacity. Its approach addresses ongoing international development and public health challenges, where assumptions about external expertise can weaken community agency and long-term results. The findings provide practical insights for global practitioners aiming to connect community cultural assets with institutional resources through participatory approaches, while promoting systems change.

### 2.2 Context

The Montagnard community in North Carolina (NC), U.S., with its five main tribes (Jarai, Rhade, Bunong, Koho, and Bahnar), represents the largest population of these indigenous groups outside Southeast Asia. Montagnards fled religious and cultural persecution in Vietnam’s Central Highlands after allying with the U.S. military during the Vietnam War. Their sophisticated cultural knowledge is often unrecognized by mainstream institutions (43, 44). Despite meaningful contributions to the region’s growing diversity, Montagnard families remain invisible within institutional data systems that track Asian American statistics. Their poor integration into mainstream society results from language barriers, low socioeconomic status, poor health literacy, cultural isolation, and limited educational access (45, 46).

The MHyP development was a genuinely community-led effort involving diverse Montagnard stakeholders such as formerly trained medical professionals, community health workers, tribal elders, MCS, and academic researchers in Greensboro, North Carolina. This city has the highest number of Montagnards in the state and is known for its civil rights history and vibrant educational scene. It comprises two renowned Historically Black Colleges and Universities (HBCUs), a minority-serving institution (MSI), two social justice-oriented liberal arts colleges (affiliated with Quakers and United Methodists), and a technical community college (35). In 2011, a Montagnard leader, a practicing doctor in Vietnam before U.S. resettlement, approached a community advocate for technical assistance with a proposed study, “The Early Hypertensive Detection Project in the American Montagnard Community.” The goal was to investigate “the hypertension problem” in the community (47). The proposed study led to several conversations in 2012 between community stakeholders and academic researchers. During these conversations, participants shared personal and professional motives, discussed tribal politics, explained intergenerational dynamics, and determined individual and collective roles and responsibilities to move the project forward (47).

Community leaders viewed their youth as bridges to a changing environment. They recognized that young people had advanced bicultural navigation skills that benefited families and trusted academic researchers (35, 47). Community leaders, who once doubted and harshly criticized youth, supported these younger members in exploring community issues and collaborating with academic researchers to plan and execute investigations. These advancements allowed researchers to introduce CBPR, emphasizing the importance of community knowledge and lived experience and how specific HIPs could help youth develop individual skills and confidence.

### 2.3 Four-phase MHyP with integrated HIPs-CBPR-approach

**Figure 2:**
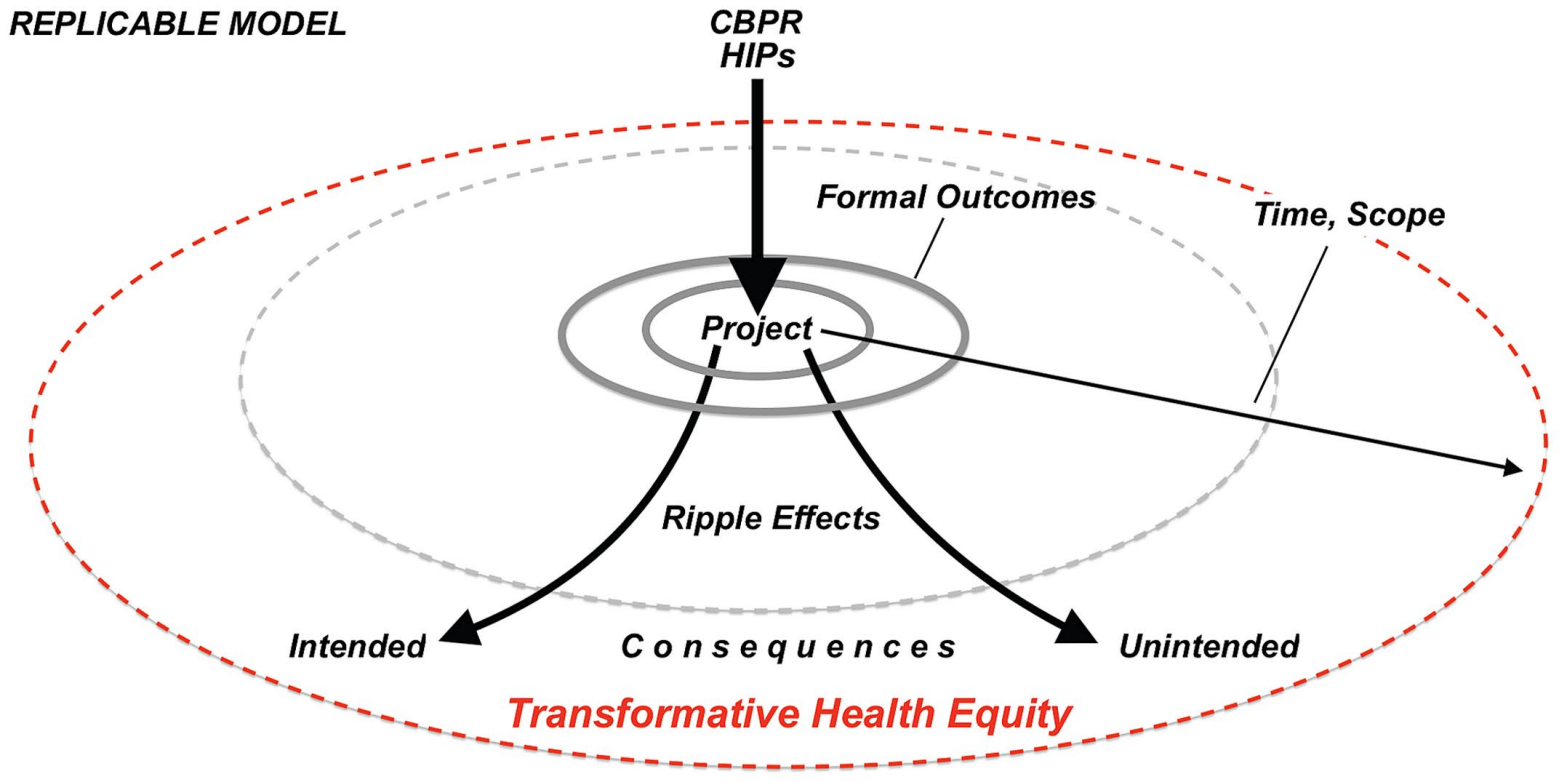
Replicable model of CBPR with HIPs ripple effects.

The MHyP received Institutional Review Board (IRB) approval in 2012 after a thorough refinement process involving academic researchers and community stakeholders to ensure informed consent procedures were culturally responsive to pre-literate and limited English-proficient (LEP) participants. From 2013 to 2017, academic researchers collaborated on a four-phase process that integrated specific HIPs in each phase (Figure 2). While MHyP engaged more than 30 college students from three local and several other distant institutions, we focus here on the MCS who served as cultural interpreters and co-researchers. They received compensation through academic credit, service-learning, work/study hours, undergraduate research assistantships, internships, and scholarships for service-learning (35). These financial compensation mechanisms reflect CBPR’s core principles of valuing community contributions, sharing institutional resources, and promoting mutual benefit among partners (27).

**Phase 1:** Terminology Development (Spring and Summer, 2013).

**HIPs:** Service Learning.

**Implementation:** MCS acted as linguistic experts alongside former Montagnard medical doctors (now serving as interpreters), community health workers, and non-MCS, creating “a dictionary of terms parallel to Montagnard/English terminology for general health and hypertension” (47). MCS matched cultural explanations of hypertension, such as “damage to blood flow,” with simplified yet accurate clinical language (35, 47).

**Phase 2:** Focus Group Discussions (Fall 2013–Spring 2014).

**HIPs:** Service Learning and Undergraduate Research.

**Implementation:** Academic researchers conducted two audio-recorded focus group discussions (FGDs) in the Jarai and Rhade tribal languages to identify concepts and themes for integration. As co-researchers, MCS designed the FGD guide using terms and concepts from the Montagnard /English dictionary and translated it into the tribal languages. They also served as bicultural/bilingual moderators between academic researchers and participants, ensuring that responses retained their linguistic meaning and cultural context (48). MCS also trained non-MCS team members in observational methods to assess participants’ interactions, reactions, and behaviors during the FGD sessions (47). Additionally, they transcribed, translated, and collaborated on analyzing transcripts and verifying thematic findings from the audio-recordings. MCS involvement enhanced rigor and validity by reducing the chance of academic researchers misinterpreting responses or missing nuanced meanings in cultural expressions (48).

**Phase 3:** Survey Design and Cognitive Testing (Spring and Fall, 2014).

**HIPs:** Service Learning, Undergraduate Research, Diversity/Global Learning.

**Implementation:** MCS identified relevant measures for chronic disease risk and profiling Montagnard’s health status through literature reviews and by extracting information from government documents and service provider websites. MCS selected culturally appropriate items and wording better suited for pre-literate participants (46, 47). They conducted cognitive testing of the questionnaire, which involved assessing comprehension and interpretation of survey questions (49), through role-play with other MCS members and feedback from non-MCS researchers. Academic researchers adjusted the questionnaires to reflect the distinct cultural communication patterns regarding the selected variables under study (46, 47).

**Phase 4:** Behavioral and Biological Data Collection (Spring 2015–Summer 2017).

**HIPs:** Service Learning, Undergraduate Research, Diversity/Global Learning, Capstone Courses and Projects, Internships.

**Implementation:** All college student researchers in the MHyP received structured training in human subjects’ protection and quantitative and qualitative research methods. MCS organized and managed data collection and analysis teams through HIPs (service learning, undergraduate research, capstone courses, honors theses, and discipline-specific internships) while fostering leadership skills. MCS also pilot-tested all data collection methods with each other and with non-MCS participants to ensure culturally sensitive, responsive, and respectful data collection (46, 47, 49). They also supported quality control measures by double-checking with non-MCS peers and collaborating with academic researchers to identify and resolve discrepancies.

### 2.4 Multi-institutional coordination strategy

The MHyP facilitated the integration of HIPs into CBPR mainly across three local institutions, establishing coordinated programs that increased MCS involvement in public health research. This model improved academic researchers’ ability to connect refugee communities with institutional resources and spaces (46, 47). Cross-institutional benefits included resource sharing (such as shared training, expertise, and programs) and expanded HIPs that recognized and rewarded MCS across institutions (like course credits, assistantships, internships, service-learning hours, etc.). MCS participated regardless of their home institutions’ community engagement capacity. The project also involved diverse college students (African American, African, Asian, Latinx, mixed-race, international, immigrant, refugee-origin, low-income, and non-traditional students), fostering peer networks within and across campuses (35). MCS accessed professional development opportunities through service learning and internship experiences (47). MCS received financial support through scholarships and travel funds to present findings at local, state, and national conferences (35). This financial support provided a tangible way to operationalize CBPR’s core principles of co-learning, mutual benefits, shared power, and capacity building for long-term community engagement (9, 27).

### 2.5 Economic support strategy

The project recognized that genuine youth engagement requires overcoming structural barriers. For example, campus offices supporting undergraduate research offered paid research assistantships to MCS for MHyP. These offices organized annual research expos where participants could showcase their results. These events provided professional development opportunities that promoted ongoing educational growth (35, 47). MCS, involved in the Bonner Foundation’s program for students from low-income families, which requires community service while attending college or university, could fulfill service hours through their involvement with MHyP (50). Faculty researchers provided career counseling and helped Montagnard youth find opportunities to improve their disciplinary skills. They also wrote recommendation letters for scholarships, graduate and professional studies, and jobs (35, 47).

### 2.6 Conflict transformation innovation

Economic pressures create conflicting demands between family survival strategies and opportunities for youth educational advancement. As a result, MCS struggle to meet their elders’ expectations and pursue their personal and academic goals (35, 37). The MHyP integrated conflict transformation methods such as dialogue, negotiation, and mediation with Diversity/Global Learning HIPs components (51–53). According to Lederach’s framework (51), dialogue encourages deep listening and mutual understanding. Faculty researchers organized social gatherings in familiar settings—mediation spaces—to promote open dialogue and deep listening between Montagnard elders and MCS, emphasizing recognition of elder contributions, and youth commitment to cultural preservation (35, 47). As trusted mentors, faculty negotiated the safe spaces for these conversations, helping engage participants without imposing outcomes (51–53).

Elders openly voiced their worries about youth losing their language and traditions. MCS responded respectfully, expressing gratitude for the elders’ sacrifices while sharing frustrations about balancing family responsibilities with career aspirations (35). The project applied these conflict transformation strategies to address future intergenerational tensions, leading to a shift in elder perceptions, viewing MCS as credible and trusted next-generation leaders (35).

## 3 Results

**Figure 3:**
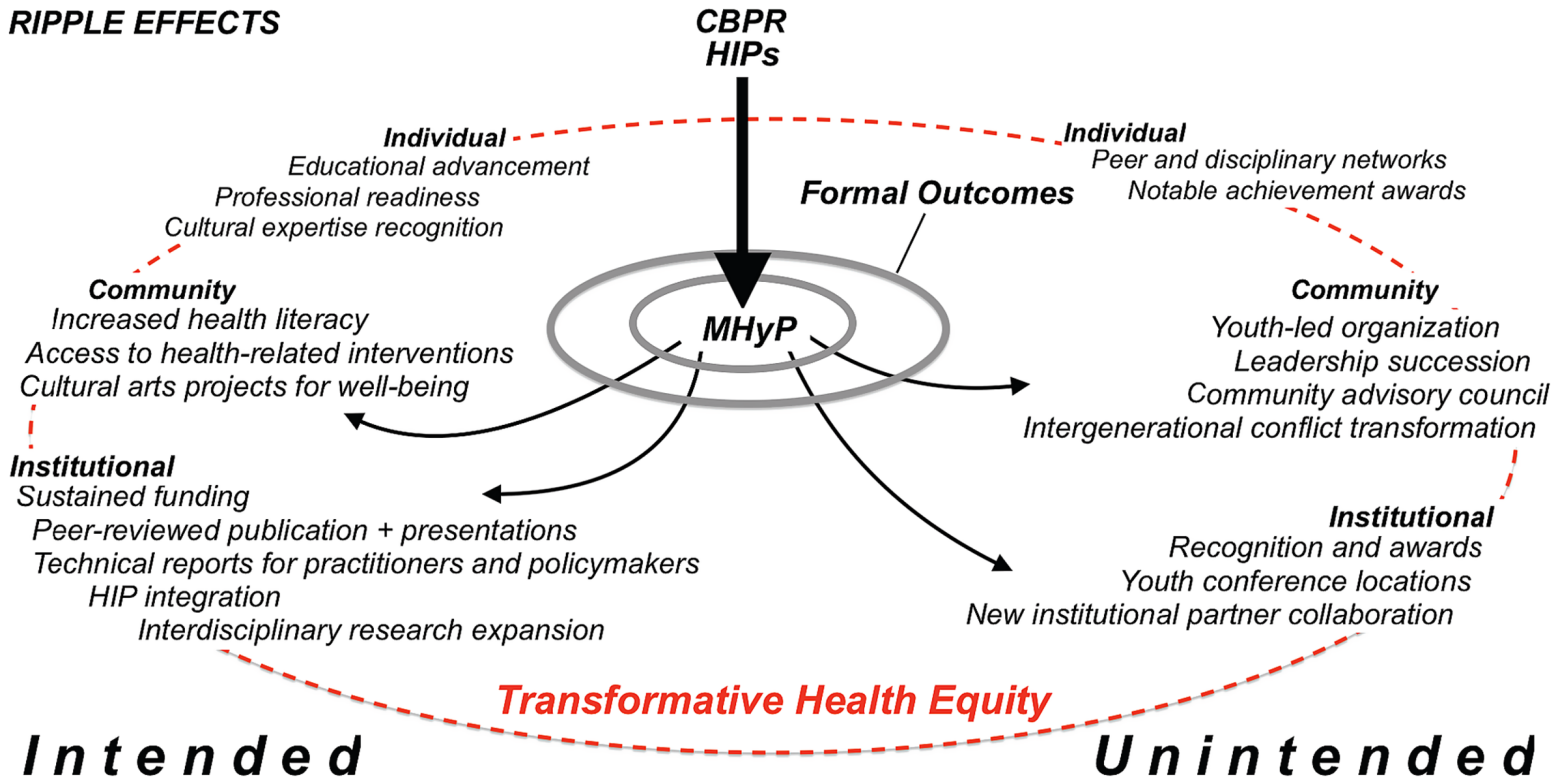
Multilevel ripple effects of MHyP.

Using HIPs within a CBPR project produced transformative outcomes at individual, community, and institutional levels that continued to influence each level long after the project ended. By applying the ripple effects framework, we show how this approach triggered cascading changes that addressed immediate health needs and structural inequities, resulting in both intended formal outcomes and unforeseen transformative impacts on health equity (Figure 3).

### 3.1 Individual level: intended

Educational Advancement: The HIPs with CBPR integration improved educational success in the Montagnard community by recognizing their MCS’ cultural expertise. Instead of seeing cultural background as a barrier, service-learning helped MCS view it as an advantage (54, 55). Undergraduate research training provided academic credentials and increased the number of graduate school applications while fostering mentorship networks that support long-term success (56, 57).

Professional Readiness: Service learning, capstone projects (honors theses and independent studies), and disciplinary internships created structured pathways for academic success and career growth after graduation, where none previously existed (58, 59). The MHyP used these HIPs to enhance capacity for student-led inquiry and community-based practice (47).

Cultural Expertise Recognition: Diversity and Global Learning components affirmed MCS’ cultural identities and personal agency, which proved essential for building confidence and group identity on campus, in the community, and in the broader world (60). MCS led innovative projects documenting and preserving community assets while earning academic recognition, demonstrating how cultural expertise can promote scholarly achievement (61, 62). CBPR and HIPs created multiple pathways for educational success while honoring cultural expertise (62).

### 3.2 Individual level: unintended

Peer and disciplinary networks: HIPs-CBPR integration across institutions established peer support networks that persisted beyond the formal project period. MCS formed professional and social relationships across disciplines and institutions, helping them enhance skills, advance their careers, and engage more in their communities. These individuals now serve as mentors to youth, role models for siblings, and future leaders in their community (35).

Notable achievement awards: recognition of cultural expertise as an academic qualification led to individual accomplishments that surpassed initial project goals and expectations. They were the “notable first recipients” of awards and prestigious fellowships, such as the four-year Bonner Scholarship for Service Learning, the Sullivan Award for distinguished community service and impact, and the inaugural Montagnard recipient of the BS in Public Health Education with a Community Health Education concentration. A youth-authored publication, “Edible Plants/Hla Arok Bong,” is the first bilingual book for the Montagnard community. This same youth conducted the first IRB-approved research on plants used for food and medicinal purposes in Vietnam (63).

### 3.3 Community level: intended

Increased health literacy: MCS developed and implemented culturally responsive health education that addressed language and cultural barriers and economic hardships, which posed significant obstacles to healthcare access (64, 65). Through Service Learning and Internship components, they created programs that integrated traditional health knowledge with modern health promotion strategies, developed health education materials on hypertension, flu, COVID, and mental health in multiple languages, and used various media such as infographics and YouTube videos suitable for pre-literate and LEP members (65, 66).

Access to health-related interventions: The project gathered baseline data that was previously unavailable. Results showed significant challenges—56% of respondents had high blood pressure, 77% were overweight, and 100% scored high on depression symptoms—and identified protective factors such as limited tobacco and alcohol use, vegetable-rich diets, and strong religious ties (46). Undergraduate research training allowed systematic documentation of health disparities and strengths (56). MCS helped facilitate family conversations about health behaviors and socioeconomic issues that were culturally difficult to discuss (46). This process improved family communication about health, healthcare decision-making, and access to disease prevention services like chronic disease screenings, flu, and COVID-19 vaccinations (46, 47).

Cultural Arts Projects for Wellbeing: Community cultural preservation through youth-led documentation initiatives increased community pride and helped maintain cultural continuity. MCS created bilingual educational materials and organized traditional arts programs that celebrated Montagnard heritage as a unique and distinct identity separate from East Asian Americans (67).

### 3.4 Community level: unintended

Youth-Led Organization: the Montagnard American Organization’s (MAO) formation in 2015 was a significant unintended community-level outcome (68). MCS from different tribal backgrounds and schools started this organization to set themselves apart from their elders’ nonprofit, the Montagnard Dega Association (MDA), to attract more community youth. HIPs-trained MAO members hosted social and health events, including youth soccer, traditional arts classes, and community health initiatives (flu vaccine clinics, COVID pandemic assistance, fundraisers) (35, 68). More recently, they purchased land and unveiled an ambitious plan for a multi-acre community center, including a health center.

Leadership succession: MAO’s creation helped build infrastructure for community advocacy and leadership that went beyond project goals. HIPs-CBPR-trained MCS led education, health, cultural preservation, and advocacy initiatives. After more than 30 years of guidance by traditional elders, MDA leadership gracefully transitioned to a former MCS trainee under the HIPs-CBPR model. MAO continues as the youth branch of MDA, now called the Montagnard Association of North America (MANC). This generational leadership transfer to youth, based on mutual trust, respect, and elder support, is a clear example of conflict transformation (47, 52). MANC/MAO has significantly enhanced the community’s health profile through COVID-19 outreach, capacity building, emergency housing aid, mental health awareness programs, and cultural arts preservation for intergenerational wellbeing, with over $1,000,000 in secured funding (68). However, the most notable unintended ripple effect has been reputational rise at the state level. MANC/MAO leadership received invitations to numerous state events, including the 2023 World Refugee Day celebration at the Governor’s mansion. MANC’s executive director was awarded the “Americans by Choice” national award. Officials invited MANC/MAO to contribute to the Governor’s report on New Americans and the NC Institute of Medicine’s (NCIOM) Immigrant Mental Health report (69, 70).

Community Advisory Council: beyond establishing the MAO, the project developed advisory structures to focus on community needs, perspectives, and concerns in research partnerships. The Community Advisory Council (CAC), formed in 2019 and whose membership includes HIPs-CBPR-trained MCS, offers guidance and cultural oversight for community-based studies (71). The CAC ensures ethical, fair, and community-centered guidelines for engaging Montagnard participants, demonstrating increased capacity for advocacy (71).

### 3.5 Institutional level: intended

Sustained funding: several internal and external mechanisms supported the MHyP, including over $40,000 in student research assistantships and more than 10,000 student service-learning hours. Five Bonner Scholars from the Montagnard community received full tuition (worth over $800,000) plus stipends. Faculty researchers secured grants from various foundations, universities, and federal programs, including Science Education for New Civic Engagements and Responsibilities (SENCER), specifically supporting HIPs integration with community engagement (72). This ongoing funding enabled continued programming beyond the initial research period.

Peer-reviewed publications and presentations: the project produced peer-reviewed publications co-authored by faculty researchers and MCS, documenting the methodology and outcomes (35, 46, 47, 66, 72). These publications contribute to academic research on community engagement and refugee health. Team members also presented findings at local, state, and national conferences, gaining recognition for the HIPs-CBPR integration model and its success with marginalized populations.

Technical reports for practitioners and policymakers: the project produced detailed documentation for practitioners and policymakers, including a needs assessment with policy recommendations (73). The materials provide evidence to support institutional policy development and program replication efforts.

HIPs integration: educational institutions recognize the importance of coordinated programming to engage LIAACRO students. Using HIPs with low-income and underserved youth offers a practical way to improve health outcomes and promote social justice (29). These changes support sustainable strategies for future community engagement while addressing barriers that prevent refugee-origin students from participating in research (30, 74).

Interdisciplinary research expansion: the project’s success has fostered the growth of community-engaged research across various disciplines and departments (biology, community and justice studies, education, human development and family studies, peace and conflict studies, psychology, public health education, nutrition, social work, and sociology), creating new opportunities for faculty and students to collaborate with community partners (35, 47).

### 3.6 Institutional level: unintended outcomes

Recognition and awards: the MHyP received unexpected institutional recognition for exemplary community engagement, earning local and national praise for outstanding CBPR projects by students and faculty. For example, the MHyP partnership was honored with the 2019 Community Outcomes and Impacts Award from the International Association for Research on Service Learning and Community Engagement (IARSLCE) (75). This recognition elevated the profile of community-engaged scholarship within participating institutions and beyond.

New institutional partner collaboration and youth conference hosts: the project’s success attracted new institutional partners interested in adopting similar community-driven research methods through the Montagnard/Asian Communities Research Network. The network was founded in 2012 by interdisciplinary researchers, advocates, professionals, and community members to promote best practices in service learning and community-engaged research, improve scholarship, and strengthen community connections. Through mutual agreement, the network moved from its university home to the MANC in 2019, where its Community Advisory Committee guides it. This shift reflects higher education’s increased acknowledgment of community expertise and leadership.

MCS guided academic researchers on other important HIPs-CBPR projects, such as oral history with older adults and the Montagnard Population Count Project. They participated in network-sponsored symposia to share insights and disseminate research findings about LIAACRO (75). Local universities and colleges hosted MAO-sponsored youth conferences on their campuses. Montagnard youth became highly sought-after speakers at these and future workshops, sharing their knowledge with broader academic and practitioner audiences.

## 4 Discussion

This community case study demonstrated how CBPR’s traditional approach of positioning community members as co-leaders was strengthened when they, through HIPs integration, could effectively access and use institutional resources and opportunities. Our findings indicate that the MHyP, the core of the HIPs-CBPR integration model, provided an innovative and replicable way to connect CBPR principles with higher education systems, i.e., bringing community members into academic settings through funded research training, service-learning, internships, coursework, and scholarships, while embedding academic researchers in the community as co-learners. This reciprocal exchange creates equitable pathways for both knowledge development and educational advancement.

In this case, the MHyP’s primary role was to transform MCS (i.e., the community members) through HIPs, turning cultural insiders into novice researchers who could use their lived experiences of poverty, social invisibility, and educational inequity to advance community health goals (54, 55). The strategic integration of five HIPs offered multiple structured pathways for community engagement tailored to different learning styles and economic situations. Each of the academic HIPs supported a CBPR principle: undergraduate research developed technical skills while encouraging shared learning and rigor; service-learning fostered reflection and mutual benefit; diversity/global learning acknowledged cultural experiences and expertise; internships in work settings, built capacity; and capstone projects promoted sustainability through leadership and community impact (28–30).

By integrating HIPs into CBPR, MCS gained access to university financial and physical resources, mentorship, and academic credentials that are often unavailable to LIAACRO youth. At the same time, non-MCS and academic researchers maintained ongoing access to community settings. This process improved cultural competence and research relevance. It created pathways for educational advancement and supported capacity building, including academic success and MCS’s professional growth. The MHyP also showed that ethical and equitable institutional resource mobilization can promote CBPR’s shared power, decision-making, capacity-building, and mutual-benefit goals (76, 77).

The ripple effects framework provided an analytical lens for documenting how empowerment evolved beyond the project’s scope and timeline. It captured short-term outcomes, such as MCS skills in data collection and professional presentations, as well as long-term effects, including community organizing, leadership development, and capacity building for sustained advocacy (78). This sustained advocacy was a result of MHyP’s layered design. MCS progressed from novice researchers to advocates who now lead initiatives focused on mental health literacy and intergenerational wellbeing—demonstrating the project’s lasting influence (79).

MCS were cultural and linguistic bridges between traditional health beliefs and modern systems through HIPs-linked CBPR experiences. Their undergraduate research and service-learning projects respected cultural protocols while tackling socioeconomic barriers to care (46, 80). These collaborations yielded rigorous, culturally responsive translational research identifying challenges (e.g., mental health stigma, limited health access) and protective assets (e.g., low substance use, healthy diets, strong faith networks) (46). By emphasizing assets, practitioners can design interventions that leverage existing strengths rather than focus on deficits, a core principle of CBPR and HIP pedagogy (81, 82). The project’s youth innovations, such as multilingual health education materials and video consent processes for pre-literate elders, demonstrate how cultural insiders can create communication strategies that external professionals rarely develop (83). These products, now shared internationally, showcase the global importance of this model for working with limited-English-proficiency (LEP) and tribal populations (84). The integration of HIPs with CBPR promoted community empowerment and institutional transformation, advancing health equity (82). CBPR anchored the academic-community partnership in principles of co-learning and mutual trust; HIPs extended CBPR’s principles by embedding education, training, leadership development, and professional readiness into every stage of the research process (85, 86). This process built a sustainable advocacy capacity and mutual access that redefined how universities and communities collaborate for health equity and social change (87).

### 4.1 Implications

A key contribution of this case study is its use of university resources to expand the access of minority communities, such as LIAACRO, to academic spaces and opportunities. These community partners gained skills, networks, and visibility through student projects and co-curricular initiatives, strengthening their presence in community, educational, and advocacy venues (88). This deliberate alignment of institutional resources with community-defined goals enhanced research quality and rigor, positioned community members as co-educators and co-investigators, and transformed traditional systems of knowledge production.

The ripple effects framework provided a systematic way to track how community empowerment and capacity-building evolved over time. Documenting both short-term and long-term outcomes, from individual leadership growth to institutional changes and advocacy, broadens the conventional success metrics. If the MHyP initiative had been narrowly defined, the replicable approach to authentic partnership revealed through these ripple effects might have been overlooked. The framework shows how evaluating processes and products can capture the depth and sustainability of CBPR outcomes (89). Ripple effects were evident across multiple outcome levels. The community-led research on LIAACRO health disparities challenged the “model minority” myth and elevated refugee-specific experiences. HIPs integration with CBPR engages students and academic researchers in meaningful, co-learning, and reflection (90, 91). At the institutional level, findings informed advocacy influencing local and university policies regarding refugee engagement and LIAACRO data disaggregation. The outcomes demonstrate how authentic partnerships can trigger simultaneous change within individuals, communities, and institutions (86).

The MHyP’s sustained influence reflects the power of aligning CBPR principles with structural supports such as diverse funding, operational frameworks for equitable participation, and leadership development that prepares community members to serve as advocates. These elements demonstrate how community empowerment and institutional transformation reinforce one another. Conceptual models like the ripple effects framework enable practitioners to evaluate immediate achievements and lasting social, academic, and policy impacts, thereby redefining how universities and communities collaborate for social change (83, 87). HIPs integration with CBPR offers a replicable pathway for public health researchers and practitioners to engage marginalized communities in global settings in ways that promote educational advancement, community values, and empowerment, and institutional transformation (92). Finally, this community case study contributes to public health’s understanding of how to reimagine and successfully deploy institutional resources—including co-curricular programming, internal funding mechanisms, and campus spaces—to support sustainable community engagement for health equity (85, 88).

### 4.2 Limitations

Traditional grant cycles often create funding gaps that threaten CBPR project partnerships. When these funds run out, community partners fear exploitation and abandonment due to past negative experiences with institutions that prioritize new research over long-term collaboration. The MHyP addressed this by adopting a phased approach and establishing a diverse CBPR ‘funding ecosystem’ that combined several small university grants with local foundation funding, along with available HIPs funding (including undergraduate research assistantship stipends, honorariums, internships, and service-learning hours that offer academic credit).

Academic schedules often conflict with community timelines. The MHyP encountered scheduling issues due to semester demands, grant deadlines, IRB approval processes, community rhythms and events, and family obligations. MCS frequently prioritized caring for elders and siblings or working extra hours over research meetings and data collection. In response, the MHyP adopted a “community-responsive scheduling” approach with flexible deadlines, multiple pathways to completion through training and cohort deployment, and aligning research activities with community events and religious services held throughout the year. This strategy helped MCS stay engaged and maintain momentum, even during busy semesters or spring and summer breaks.

## 5 Conclusion

In conclusion, integrating HIPs with CBPR offers a proven and reproducible model for equitable partnerships and mutual benefits between LIAACRO and higher education institutions. The model’s innovation lies in expanding community access to academic resources and spaces that would otherwise be inaccessible. It also enhances institutional responsiveness. Supported by the ripple effects framework, the intentional alignment of HIPs’ educational infrastructure with CBPR’s core principles of co-learning and capacity-building results in outcomes such as skill and leadership development, empowerment, and sustained advocacy. The HIPs-CBPR model provides a transferable strategy for global health practitioners to promote health equity and community advocacy within refugee populations.

## Data Availability

The raw data supporting the conclusions of this article will be made available by the authors upon reasonable request.
